# Multiple Suboptimal Solutions for Prediction Rules in Gene Expression Data

**DOI:** 10.1155/2013/798189

**Published:** 2013-04-16

**Authors:** Osamu Komori, Mari Pritchard, Shinto Eguchi

**Affiliations:** ^1^The Institute of Statistical Mathematics, Midori-cho, Tachikawa, Tokyo 190-8562, Japan; ^2^CLC Bio Japan, Inc., Daikanyama Park Side Village 204, 9-8 Sarugakucho, Shibuya-ku, Tokyo 150-0033, Japan

## Abstract

This paper discusses mathematical and statistical aspects in analysis methods applied to microarray gene expressions. We focus on pattern recognition to extract informative features embedded in the data for prediction of phenotypes. It has been pointed out that there are severely difficult problems due to the unbalance in the number of observed genes compared with the number of observed subjects. We make a reanalysis of microarray gene expression published data to detect many other gene sets with almost the same performance. We conclude in the current stage that it is not possible to extract only informative genes with high performance in the all observed genes. We investigate the reason why this difficulty still exists even though there are actively proposed analysis methods and learning algorithms in statistical machine learning approaches. We focus on the mutual coherence or the absolute value of the Pearson correlations between two genes and describe the distributions of the correlation for the selected set of genes and the total set. We show that the problem of finding informative genes in high dimensional data is ill-posed and that the difficulty is closely related with the mutual coherence.

## 1. Introduction

The Human Genome Project [1, 2] has driven genome technology forward to exhaustive observation. The accumulation of genome knowledge leads us to study gene and protein expressions to elucidate the functions of genes and the interaction among genes. We overview the progress of microarray technology for gene expressions and the analysis methods based on gene expression towards good prediction for phenotypes. Analysis of gene expressions has been rapidly developed and enhanced by microarray technology. In the current stage, this progress enables us to observe all the gene expressions of subjects in an exhaustive manner. It is opening an area of bioinformatics to discover the relation of phenotypes with gene expressions, where phenotypes imply degrees and stages of pathological response, treatment effect and prognosis of patients. We anticipated that a breakthrough in medical and clinical science will lead to the discovery of key understandings which elucidate the associations between phenotypes and gene expressions. For this, the machine learning approach is successfully exploited including support vector machine, boosting learning algorithm, and the Bayesian network.

However there exists a difficult problem in analyses for gene expression data in which the number of genes is much more than that of samples. Such an extreme unbalance between the data dimension and sample size is a typical characteristic in genomics and omics data; this tendency will become more apparent on account of new technology in the near future. Appropriate methods for solving the problem are urgently required; however, there are severe difficulties with attaining a comprehensive understanding of the phenotypes based on the set of exhaustive gene expressions. We face many false positive genes sinking true positive genes in the prediction, which creates an impediment to building individualized medicines. There are a vast number of proposals with complex procedures to challenge the difficult problem of extracting robust and exhaustive information from gene expressions. Sparse type of feature selection is one example; it is considered to avoid overfitting and obtaining an interpretable model for gene expression data. In the regression context, Tibshirani [3] proposed Lasso which achieved feature selection by shrinking some coefficients to exactly zero. In the image processing area, the sparse model is also considered for redundant representation of data by Donoho and Elad [4] and Candès et al. [5].

The current status for prediction performance has attained a constant level; however, there are still unsolved problems in the prediction in which the observed number of genes is extremely larger than that of the subjects. This causes difficulties in which superfluous discovered genes arise the expressions which have weak power for prediction. As a result, almost all microarray data analysis is not completely confirmed as the biological replication which is discussed in Allison et al. [6].

We address the microarray data analysis discussed in van't Veer et al. [7], in which a set of 70 genes is selected for prediction for prognosis in breast cancer patients as informative biomarkers. The result helps to build the prediction kit named “MammaPrint” which was approved by the FDA in 2007. We make a reanalysis of the data and discuss how surprisingly many other gene sets show almost the same prediction performance as the gene set published. Thus, their gene set does not uniquely show a reasonable power of prediction; so we suggest that it is impossible to build up a universal prediction rule by efficient selection of genes. In particular the ranking procedure according to association measure to the phenotype is very fragile for data partition. We discuss the statistical reason why the data analysis of gene expression is involved with such difficulties of multiple solutions for the problem. We calculate the value of mutualcoherence for genes in MammaPrint and show the essential difficulty of determining informative genes among huge number of genes used in the data analysis.

This paper is organized as follows. In Section 2, we describe the pattern recognition of gene expression and overview the current proposed methods. In Section 3, we point out the current difficulty in gene expression using a real data set. Finally, we discuss the results and future works in Section 4.

## 2. Pattern Recognition from Gene Expressions

 In this section, we overview the pattern recognition of gene expression. First we mention a DNA microarray technique which is the widely used method to measure millions of gene expressions. Then we present the current methods for gene selection using microarray.

### 2.1. DNA Microarray

Microarray has become a widely used technology in a variety of areas. Microarray measures the amount of mRNA or gene expression. There are two major technologies available for gene expression measurement. One is GeneChip system provided by Affymetrix Inc. GeneChip uses prefabricated short lengths of oligonucleotide. The other is cDNA array which was originally developed by Schena et al. [9]. We briefly mention both technologies.

GeneChip uses a pair of short length oligonucleotides attached to a solid surface, such as glass, plastic or silicon. The short pair of oligonucleotides is called the probe pair. Each probe pair is composed of a perfect match (PM) probe and a mismatch (MM) probe. PM is a section of the mRNA of interest, and MM is created by changing the middle (13th) base of the PM with the intention of measuring nonspecific binding. mRNA samples are collected from subjects such as cancer patients then labeled with fluorescence dye. If the short oligonucleotide is matched with the mRNA sample, the labeled mRNA sample is hybridized to a spot of microarray. If the labeled mRNA and the probe match perfectly, they bind strongly otherwise they bind weakly. Those with weak or nonspecific binding are washed out by a washing buffer; then only strongly bound mRNA samples are measured by a scanner. Scanned measurements need further processing before analysis such as outlier detection, background subtraction, and normalization. These processes are called preprocessing.

In the early stage of microarray, the quality of microarray measurements contained a lot of variance. Therefore, pre-processing was a very active research area. Affymetrix recommended the use of both PM and MM probes to subtract nonspecific binding and implement MASS algorithm to their software; however, Irizarry et al. [10] and Naef et al. [11] pointed out that the normalization model considering MM captures nonspecific effect more than reality. Currently robust multichip average (RMA) which is introduced by Irizarry et al. [10] is also widely used.

cDNA array uses glass slides to attach short oligonucleotides probes. cDNA array uses inkjet printing technology. GeneChip uses one color fluorescent dye, on the other hand, cDNA array utilizes two different color fluorescent dyes. One of the colors is for controlling mRNA, and the other color is for treatment of mRNA. Both samples are hybridized on the same array. The scanner detects both fluorescent dyes separately. Data processing is slightly different from GeneChip. As cDNA uses two fluorescent dyes, scanned data is normally treated as ratio data of treatment over control.

Microarray technology has improved in the last decades including reduction of the variance; normalization procedures do not have as great an effect as before. Research interest has moved to areas such as data analysis finding subclass or predicting the subclass.

### 2.2. Review for Prediction via Microarray

 The initial approach employed for subclass discovery was hierarchical clustering analysis. Golub et al. [12] showed the result of clustering for leukemia data using microarray. In their result, subclasses of leukemia were well clustered by gene expression pattern. This result was hailed as a new dawn in the cancer classification problem.

Breast cancer is one of the most used cancers for the gene expression classification problem. Breast cancer treatment decisions are based largely on clinicopathological criteria such as tumor size, histological grade, and lymph node metastatsis; however, van't Veer et al. [7] pointed that the majority of patients received unnecessary chemotherapy and that there is a need to find a better criteria who benefits from chemotherapy. 

van't Veer et al. [7] proposed 70 genes to predict patient outcome as first multigene signatures in breast cancer. 70 genes were decided using 78 patients' tumor samples. In brief, they selected 5000 significant expressed genes from 25,000 genes on microarray; then coefficient correlation with outcome was calculated for each class. Genes were sorted by correlation coefficient and further optimized from the top ranked gene with sequentially adding from a subset of five genes. The top 70 genes were proposed as outcome predictors. Paik et al. [13] proposed 21 multigene signatures based on RT-PCR results. These two multigene prognostic signatures are available as clinical test named MammaPrint and Oncotype DX. FDA cleared the MammaPrint test in 2007 and it is currently being tested in the Microarray In Node-negative and 1–3 positive lymph-node Disease may Avoid ChemoTherapy (MINDACT) for further assessment which is described by Cardoso et al. [14].

Besides these two multigene prognostic signatures, different multigenes were selected as prognostic signatures. Fan et al. [15] discussed a different set of multigene signatures in the breast cancer prognostic classification studies. Those signatures show little overlap; however, they still showed similar classification power. Fan et al. [15] suggested that these signatures are probably tracking a common set of biological phenotypes. Considering thousands and millions of genes on microarray, multiple useful signature sets are not difficult to imagine; however, finding a stable and informative gene with high classification accuracy is of interest.

### 2.3. Feature Selection

 Reduction of dimension size is necessary as superfluous features can cause overfitting and interpretation of classification model becomes difficult. Reducing dimension size while keeping relevant features is important. There are some feature selection methods proposed. Saeys et al. [8] provided a taxonomy of feature selection methods and discussed their use, advantages, and disadvantages. They mentioned the objectives of feature selection (a) to avoid overfitting and improve model performance, that is, prediction performance in the case of supervised classification and better cluster detection in the case of clustering, (b) to provide faster and more cost-effective models, and (c) to gain a deeper insight into the underlying processes that generated the data.  Table 1 provides their taxonomy of feature selection methods. In the context of classification, feature selection methods are organized into three categories: filter methods, wrapper methods, and embedded methods. Feature selection methods are categorized depending on how they are combined with the construction of a classification model.

Filter methods calculate statistics such as t-statistics then filter out those which do not meet the threshold value. Advantages of filter methods are easy implementation, computational simplicity, and speed. Filter methods are independent of classification methods; therefore, different classifiers can be used. Two of the disadvantages of filter methods are that they ignore the interaction with the classification model and most of the proposed methods are univariate.

Whereas filter techniques treat the problem of finding a good feature subset independently of the model selection step, wrapper methods embed the model hypothesis search within the feature subset search such as sequential backward selection [16]. Wrapper methods search all feature subsets, the feature subset space grows exponentially with the number of features. One advantage of wrapper methods is the interaction between feature subset search and model selection. Computational burden is one of the disadvantages of this approach, especially if building the classifier has a high computational cost.

The third class of feature selection is embedded techniques. Like the wrapper methods, embedded techniques search for an optimal subset of features with classifier construction. For example, SVM-RFE proposed by Guyon et al. [17]. Thus, embedded approaches are specific to a given learning algorithm. The difference from wrapper methods is that embedded techniques are guided by the learning process. Whereas wrapper methods search all possible combinations of gene sets, embedded techniques search for the combination based on a criteria. This enables reduction in computational burden.

Besides these methods, the idea of sparseness was recently introduced in some feature selection methods. One approach is to use penalties in the regression context. Tibshirani [3] proposed Lasso which uses *L*_1_ norm penalties. The combination of *L*_1_ penalty and *L*_2_ penalty is called elastic net [18]. These methods are focusing on reducing features to avoiding overfitting and better interpretability as biologists expect to obtain biological insight from selected features.

However, these sparseness ideas do not take into account multiple solutions in one data set. When the data dimension is thousands or millions, there are multiple possible solutions. Sorting genes based on some criteria; then selecting a subset from the top is not always the best selection. We elaborate multiple solutions in the following section and give some idea of how to select the optimum solution. Here, we refer to an optimal solution as a prediction rule with high classification accuracy for various data sets.

## 3. Multiple Solutions for Prediction Rules

 The existence of multiple solutions for prediction of disease status based on breast cancer data [7] was shown by Ein-Dor et al. [19], where they suggest three reasons for this problem. The first is that there are many genes correlated with disease status; the second one is that the differences of the correlation among the genes are very small; the last one is that the value of the correlation is very sensitive to the sample used for the calculation of the correlation. In the paper, they demonstrate gene ranking based on the correlation and show that there exist many equally predictive gene sets. 

In this section, we investigate the existence of multiple solutions from different viewpoints. At first, to check the variability of prediction accuracy based on different statistical methods, we apply the van't Veer method [7], the Fisher linear discriminant analysis [20], AdaBoost [21], and AUCBoost [22]. The last two methods are called boosting in machine learning community, where genes are nonlinearly combined to predict the disease status. Second, we apply hierarchal clustering to examine the heterogeneity of gene expression patterns. Sørlie et al. [23] showed there exist subtypes of breast cancers, for which the patterns of gene expression are clearly different, and the disease statuses are also different in accordance with them. Ein-Dor et al. [19] suggests that the heterogeneity of the subtypes is one reason why there are so many solutions for the prediction or large fluctuations of genes selected for the predictions. Hence, we calculate Biological Homogeneity Index (BHI) to see the clustering performance for various gene sets and examine the existence of the subtypes. Finally, we consider the mutualcoherence for the breast cancer data and discuss the relation to the multiple solutions.

The breast cancer data consists of the expression data of 25000 genes and 97 cancer patients. After a filtering procedure, 5420 genes are identified to be significantly regulated, and we focused on this filtered data. The patients are divided into a training sample (78 patients) and a test sample (19 patients) in the same way as the original paper [7]. Here, we consider classical methods and boosting methods to predict whether the patient has good prognosis (free of disease after initial diagnosis at least 5 years) or has bad prognosis (distant metastases within 5 years). The prediction rule is generated by the training sample, and we measure the prediction accuracy based on the test sample. 

### 3.1. Classification

 We briefly introduce the statistical methods used for the prediction of the disease status. The first two methods are linear discriminant function, where the genes are linearly combined to classify the patients into a good prognosis group or a bad prognosis group. The last two ones are boosting methods, where the genes are nonlinearly combined to generate the discriminant functions. 

#### 3.1.1. The van't Veer Method

 Let *y* be a class label indicating disease status such as *y* = −1 (good prognosis) and *y* = 1 (metastases), and let **x**( = (*x*_1_,…, *x*_*p*_)^*T*^) be a *p*-dimensional covariate such as gene expression. We denote the samples of *y* = −1 and *y* = 1 as {**x**_*i*_^−^ : *i* = 1,…, *n*_−_} and {**x**_*j*_^+^ : *j* = 1,…, *n*_+_}, respectively, and the mean value of the patients with good prognosis as

(1) $$\begin{array}{c} {\hat{\mu }}_{-}={\left({\hat{\mu }}_{1}^{-},\ldots ,{\hat{\mu }}_{p}^{-}\right)}^{T}=\frac{1}{n_{-}}\sum_{i=1}^{n_{-}}x_{i}^{-}. \end{array}$$

Then van't Veer et al. [7] proposed a discriminant function *F*(**x**) based on the correlation to the average good prognosis profile above, which is given as

(2) $$\begin{array}{c} F\left(x\right)=-\frac{\sum_{k=1}^{p}\left(x_{k}-\tilde{x}\right)\left({\hat{\mu }}_{k}^{-}-{\tilde{\mu }}_{-}\right)}{\sqrt{\sum_{k=1}^{p}{\left(x_{k}-\tilde{x}\right)}^{2}}\sqrt{\sum_{k=1}^{p}{\left({\hat{\mu }}_{k}^{-}-{\tilde{\mu }}_{-}\right)}^{2}}}, \end{array}$$

where

(3) $$\begin{array}{c} \tilde{x}=\frac{1}{p}\sum_{k=1}^{p}x_{k},\;\;{\tilde{\mu }}_{-}=\frac{1}{p}\sum_{k=1}^{p}{\hat{\mu }}_{k}^{-}. \end{array}$$

If the value of *F*(**x**) is smaller than a predefined threshold value, then the patient is judged to have good prognosis (*y* = −1), otherwise to have metastases (*y* = 1). This method is called one-class classification, where it focuses on only one-class label information to predict the disease status *y*. This idea is also employed in machine learning community. See Yousef et al. [24] and Gardner et al. [25] for applications in biology and medicine. 

#### 3.1.2. DLDA

 We consider Fisher's linear discriminant analysis [20], which is widely used in many applications. Suppose that **x** is distributed as *N*(***μ***_−_, Σ_−_) for *y* = −1 and as *N*(***μ***_+_, Σ_+_) for *y* = 1. Then, if Σ_−_ = Σ_+_ = Σ, the estimated log-likelihood ratio is given as

(4) F(x)=(μ^+−μ^−)TΣ^−1x−12μ^+TΣ^−1μ^+ +12μ^−TΣ^−1μ^−+log⁡n+n−,

where ${\hat{\mu }}_{+}$, ${\hat{\mu }}_{-}$ and $\hat{\Sigma }$ are the sample means for *y* ∈ {−1,1} and a total sample variance, respectively. For simplicity, we take the diagonal part of $\hat{\Sigma }$ (Diagonal Linear Discriminant Analysis) and predict the disease status based on the value of the log-likelihood ratio above. This modification is often used in a situation where *p* is much larger than *n* ( = *n*_−_ + *n*_+_). In that case, the inverse of $\hat{\Sigma }$ cannot be calculated. 

#### 3.1.3. AdaBoost

 We introduce a famous boosting method in machine learning community. The key concept of boosting is to construct a powerful discriminant function *F*(**x**) by combining various weak classifiers *f*(**x**) [26]. We employ a set *ℱ* of decision stumps as a dictionary of weak classifiers. Here the decision stump for the *k*th gene expression *x*_*k*_ is defined as a simple step functions such as

(5) fk(x)={1if  xk≥bk−1otherwise,

where *b*_*k*_ is a threshold value. Accordingly, it is known that *f*_*k*_(**x**) is the simplest classifier in the sense that *f*_*k*_(**x**) neglects all other information of gene expression patterns than that of one gene *x*_*k*_. However, by changing the value of *b*_*k*_ for all genes (*k* = 1,…, *p*), we have *ℱ* that contains exhaustive information of gene expression patterns. We attempt to build a good discriminant function *F* by combining decision stumps in *ℱ*.

The typical one is AdaBoost proposed by Schapire [21], which is designed to minimize the exponential loss for a discriminant function *F* as

(6) $$\begin{array}{c} L_{\exp}\left(F\right)=\frac{1}{n}\sum_{i=1}^{n}\exp\left\{-y_{i}F\left(x_{i}\right)\right\}, \end{array}$$

where the entire data set is given as {(**x**_*i*_, *y*_*i*_) | *i* = 1,…, *n*}. The exponential loss is sequentially minimized by the following algorithm.

(1) Initialize the weight of *x*_*i*_ for *i* = 1,…, *n* as *w*_1_(*i*) = 1/*n*.
(2) For the iteration number *t* = 1,…, *T*
  (a) choose *f*_*t*_ as
    (7) $$\begin{array}{c} f_{t}=\underset{f\in ℱ}{\text{argmin}}\;\epsilon_{t}\left(f\right), \end{array}$$
    where
    (8) $$\begin{array}{c} \epsilon_{t}\left(f\right)=\sum_{i=1}^{n}I\left(f\left(x_{i}\right)\neq y_{i}\right)\frac{w_{t}\left(i\right)}{\sum_{i=1}^{n}w_{t}\left(i\right)}, \end{array}$$
    and *I* is the indicator function,
  (b) calculate the coefficient as
    (9) $$\begin{array}{c} \beta_{t}=\frac{1}{2}\log\frac{1-\epsilon_{t}\left(f_{t}\right)}{\epsilon_{t}\left(f_{t}\right)}, \end{array}$$
  (c) update the weight as
    (10) $$\begin{array}{c} w_{t+1}\left(i\right)=w_{t}\left(i\right)\exp\left\{-y_{i}\beta_{t}f_{t}\left(x_{i}\right)\right\}, \end{array}$$
(3) output the final function as *F*(**x**) = ∑_*t*=1_^*T*^ *β*_*t*_ *f*_*t*_(**x**).

Here, we have

(11) Lexp⁡(F+βf) =1n∑i=1nexp⁡{−yiF(xi)}exp⁡{−βyif(xi)}

(12) =1n∑i=1nexp⁡{−yiF(xi)}   ×[eβI(f(xi)≠yi)+e−βI(f(xi)=yi)]

(13) $$\begin{array}{c} =L_{\exp}\left(F\right)\left\{e^{\beta }\epsilon \left(f\right)+e^{-\beta }\left(1-\epsilon \right)\right\} \end{array}$$

(14) $$\begin{array}{c} \geq 2L_{\exp}\left(F\right)\sqrt{\epsilon \left(f\right)\left(1-\epsilon \left(f\right)\right)}, \end{array}$$

where *ϵ*(*f*) = 1/*n*∑_*i*=1_^*n*^ *I*(*f*(**x**_*i*_) ≠ *y*_*i*_)exp⁡{−*y*_*i*_ *F*(**x**_*i*_)}/*L*_exp⁡_(*F*) and the equality in (14) is attained if and only if

(15) $$\begin{array}{c} \beta =\frac{1}{2}\log\frac{1-\epsilon \left(f\right)}{\epsilon \left(f\right)}. \end{array}$$

Hence, we can see that the exponential loss is sequentially minimized in the algorithm. It is easily shown that

(16) $$\begin{array}{c} \epsilon_{t+1}\left(f_{t}\right)=\frac{1}{2}. \end{array}$$

That is, the weak classifier *f*_*t*_ chosen at the step *t* is the worst element in *ℱ* in the sense of the weighted error rate in the step *t* + 1. 

#### 3.1.4. AUCBoost with Natural Cubic Splines

 The area under the ROC curve (AUC) is widely used to measure classification accuracy [27]. This criterion consists of the false positive rate and true positive rate, so it evaluates them separately in contrast to the commonly used error rate. The empirical AUC based on the samples {**x**_*i*_^−^ : *i* = 1,…, *n*_−_} and {**x**_*j*_^+^ : *j* = 1,…, *n*_+_} is given as

(17) $$\begin{array}{c} \bar{\text{AUC}}\left(F\right)=\frac{1}{n_{-}\;n_{+}}\sum_{i=1}^{n_{-}}\sum_{j=1}^{n_{+}}H\left(F\left(x_{j}^{+}\right)-F\left(x_{i}^{-}\right)\right), \end{array}$$

where *H*(*z*) is the Heaviside function: *H*(*z*) = 1 if *z* ≥ 0 and H(*z*) = 0 otherwise. To avoid the difficulty to maximize the nondifferential function above, an approximate AUC is considered by Komori [28] as

(18) $$\begin{array}{c} {\bar{\text{AUC}}}_{\sigma }\left(F\right)=\frac{1}{n_{-}\;n_{+}}\sum_{i=1}^{n_{-}}\sum_{j=1}^{n_{+}}H_{\sigma }\left(F\left(x_{j}^{+}\right)-F\left(x_{i}^{-}\right)\right), \end{array}$$

where *H*_*σ*_(*z*) = Φ(*z*/*σ*), with Φ being the standard normal distribution function. A smaller scale parameter *σ* means a better approximation of the Heaviside function *H*(*z*). Based on this approximation, a boosting method for the maximization of AUC as well as the partial AUC is proposed by Komori and Eguchi [22], in which they consider the following objective function:

(19) AUC¯σ,λ(F)=1n− n+∑i=1n−∑j=1n+Hσ(F(xj+)−F(xi−))−λ∑k=1p∫{Fk′′(xk)}2dxk,

where *F*_*k*_′′(*x*_*k*_) is the second derivative of the *k*th component of *F*(**x**) and *λ* is a smoothing parameter that controls the smoothness of *F*(**x**). Here, the set of weak classifiers is given as

(20) $$\begin{array}{c} ℱ=\left\{f\left(x\right)=\frac{N_{k,l}\left(x_{k}\right)}{Z_{k,l}}\;|\;k=1,2,\ldots ,p,\;l=1,2,\ldots ,m_{k}\right\}, \end{array}$$

where *N*_*k*,*l*_ is a basis function for representing natural cubic splines and *Z*_*k*,*l*_ is a standardization factor. Then the relationship

(21) $$\begin{array}{c} \underset{\sigma ,\lambda ,F}{\max}{\bar{\;\text{AUC}}}_{\sigma ,\lambda }\left(F\right)=\underset{\lambda ,F}{\max}\;{\bar{\text{AUC}}}_{1,\lambda }\left(F\right) \end{array}$$

allows us to fix *σ* = 1 without loss of generality and have the following algorithm.

(1) Start with *F*_0_(**x**) = 0.
(2) For *t* = 1,…, *T*
  (a) update *β*_*t*−1_(*f*) to *β*_*t*_(*f*) with a one-step Newton-Raphson iteration,
  (b) find the best weak classifier *f*_*t*_
    (22) $$\begin{array}{c} f_{t}=\underset{f}{\text{argmax}}\;{\bar{\text{AUC}}}_{1,\lambda }\left(F_{t-1}+\beta_{t}\left(f\right)f\right), \end{array}$$
  (c) update the score function as
    (23) $$\begin{array}{c} F_{t}\left(x\right)=F_{t-1}\left(x\right)+\beta_{t}\left(f_{t}\right)f_{t}\left(x\right). \end{array}$$
(3) Output *F*(**x**) = ∑_*t*=1_^*T*^ *β*_*t*_(*f*_*t*_)*f*_*t*_(**x**).

The value of the smoothing parameter *λ* and the iteration number *T* is determined at the same time by the cross validation. 

### 3.2. Clustering

 We applied a hierarchical clustering using breast cancer data [7], where the distances between samples and genes are determined by the correlation and complete linkage was applied as the agglomeration method. To measure the performance of the clustering, we used biological homogeneity index (BHI) [29], which measures the homogeneity between the cluster *𝒞* = {*C*_1_,…, *C*_*K*_} and the biological category or subtype *ℬ* = {*B*_1_,…, *B*_*L*_},

(24) $$\begin{array}{c} \text{BHI}\left(𝒞,ℬ\right)=\frac{1}{K}\sum_{k=1}^{K}\frac{1}{n_{k}\left(n_{k}-1\right)}\sum_{i\neq j,i,j\in C_{k}}I\left(B^{\left(i\right)}=B^{\left(j\right)}\right), \end{array}$$

where *B* ^(*i*)^ ∈ *ℬ* is the subtype for the subject *i* and *n*_*k*_ is the number of subjects in *C*_*k*_. This index is upper bounded by 1 meaning the perfect homogeneity between the clusters and the biological categories. We calculated this index for the breast cancer data to investigate the relationship between the hierarchical clustering and biological categories such as disease status (good prognosis or metastases) and hormone status: estrogen receptor (ER) status and progesterone receptor (PR) status. The hormone status is known to be closely related with the prognosis of the patients [23]. 

### 3.3. Mutualcoherence

 Now, we have a data matrix **X** with *n* rows (*n* patients) and *p* columns (*p* genes). We assumed an *n*-dimensional vector **b** indicating the true disease status, where the positive values correspond to metastases and negative ones to good prognosis. The magnitude of **b** denotes the level of disease status. Then, the optimal linear solution **β** ∈ ℝ^*p*^ should be satisfied

(25) $$\begin{array}{c} X\beta =b. \end{array}$$

Note that if *p* is much larger than *n*, then only a few elements of **β** should be non-zeros, and the others to be zero, which means **β** has a sparse structure. The sparsity has a close relationship with mutualcoherence [30], which is defined for the data matrix **X** as

(26) $$\begin{array}{c} \mu \left(X\right)=\underset{1\leq i,j\leq p,i\neq j}{\max}\frac{\left|x_{i}^{T}x_{j}\right|}{{||x_{i}||}_{2}{||x_{j}||}_{2}}, \end{array}$$

where **x**_*i*_ ∈ ℝ^*n*^ denotes the *i*th column in **X** or *i*th gene in the breast cancer data; |·| is the absolute value and ||·||_2_ is the Euclidean norm. This index measures the distance of columns (genes) in the data matrix **X** and is bounded as 0 ≤ *μ*(**X**) ≤ 1. The next theorem shows the relationship between sparsity of **β** and the data matrix **X**.

**Theorem 1 (Elad [30])**

If **X** **β** = **b** has a solution **β** obeying ||**β**||_0_ < (1 + 1/*μ*(**X**))/2, this solution is necessarily the sparsest possible, where ||**β**||_0_ denotes the number of the nonzero components of **β**.

This theorem suggests that the linear discriminant function *F*(**x**) = **β** ^*T*^ **x** could have a sparse solution to predict the disease status **b**, which corresponds to metastases or good prognosis in the breast cancer data. If the value of *μ*(**X**) is nearly equal to 1, then ||**β**||_0_ could become approximately 1, indicating that just one gene (a column in **X**) could be enough to predict the disease status if there were a solution **β**. This indicates that we could have a chance to find the multiple solutions with fewer genes than 70 genes in MammaPrint. Although the framework in (25) is based on a system of linear equations and does not include random effects as seen in the classification problems we deal with, Theorem 1 is indicative in the case where the number of covariates *p* is much larger than the observation number *n*. 

### 3.4. Results

 We prepare various data sets using a training sample (78 patients) based on 230 genes selected by van't Veer et al. [7] in order to investigate the existence of the multiple solutions of the prediction of the disease status, which are given by

(27) $$\begin{array}{c} 𝒟=\left\{D_{1–70},D_{6–75},\ldots ,D_{161–230}\right\}. \end{array}$$

**Figure 1:**
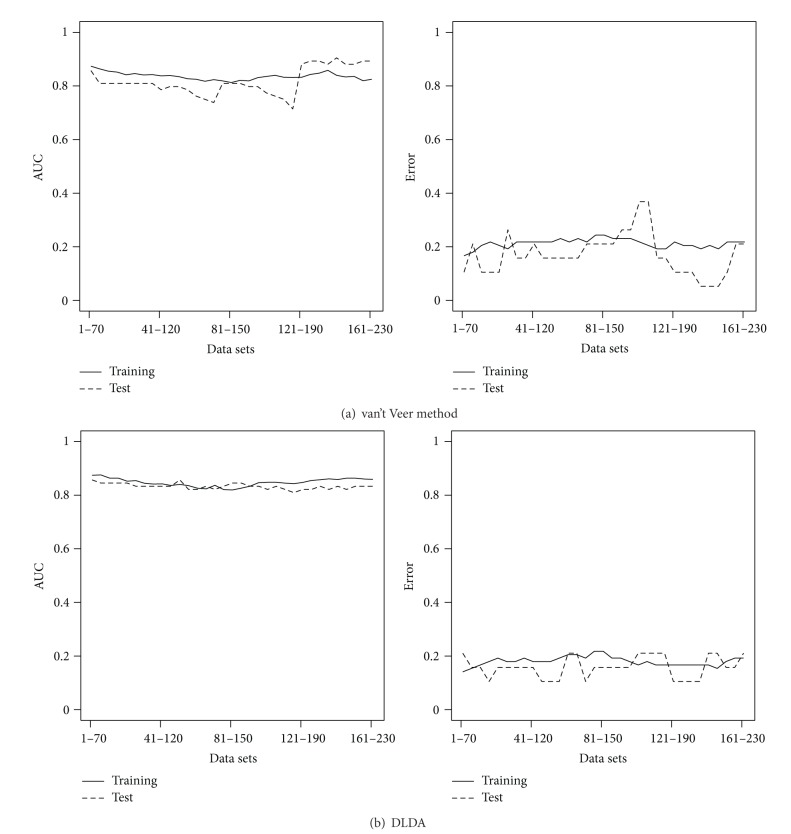
Results of classical methods. (a) and (b) show the AUC values (left panel) and the error rate (right panel) over data sets *𝒟* for van't Veer method and DLDA.

**Figure 2:**
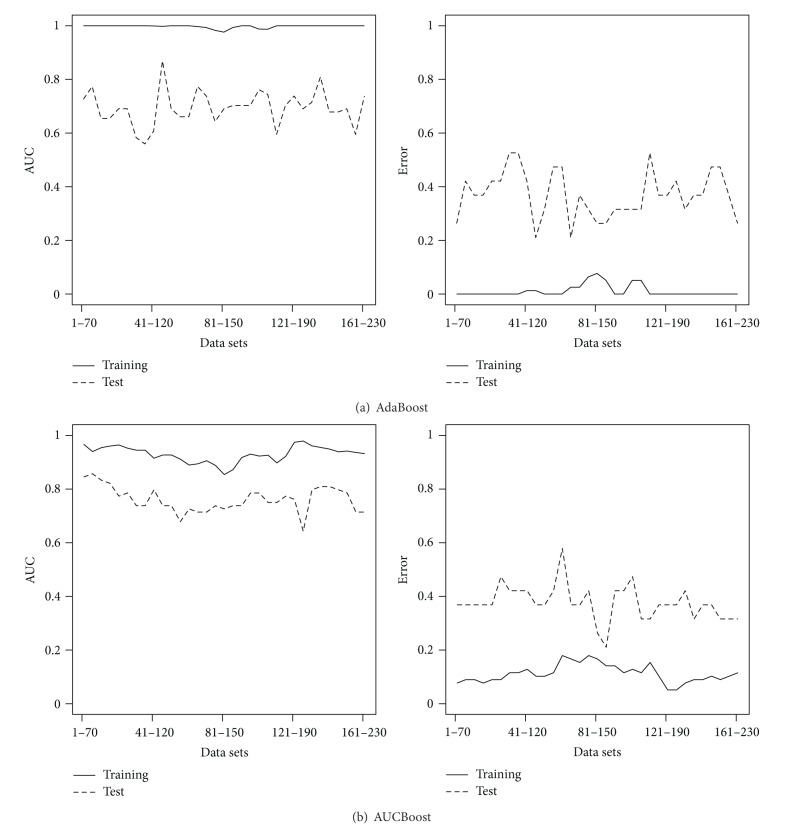
Results of boosting methods. (a) and (b) show the AUC values (left panel) and the error rate (right panel) over data sets *𝒟* for AdaBoost and AUCBoost.

The first data set *D*_1–70_ consists of 78 patients and 70 genes in MammaPrint. The ranking of the 230 genes in *𝒟* is on the basis of correlation with disease status as in [7]. We apply the van't Veer method, DLDA, AdaBoost, and AUCBoost as explained in the previous subsection to the training data to have the discriminant functions *F*(**x**), where each threshold is determined so that the training error rate is minimized. Then, we evaluate the classification accuracy based on the training data as well as the test data. The results of classification performance of van't Veer method and DLDA are illustrated in Figure 1; those of AdaBoost and AUCBoost are in Figure 2. The AUC and the error rate are plotted against *𝒟*. In regard to the performance based on the training data denoted by solid line, the boosting methods are superior to the classical methods. However, comparison based on the test data, DLDA shows good performance for almost all the data sets in *𝒟*, having the AUC more than 0.8 and the error rates less than 0.2 in average. This evidence suggests there exist many sets of genes having almost the same classification performance as that of MammaPrint.

**Figure 3:**
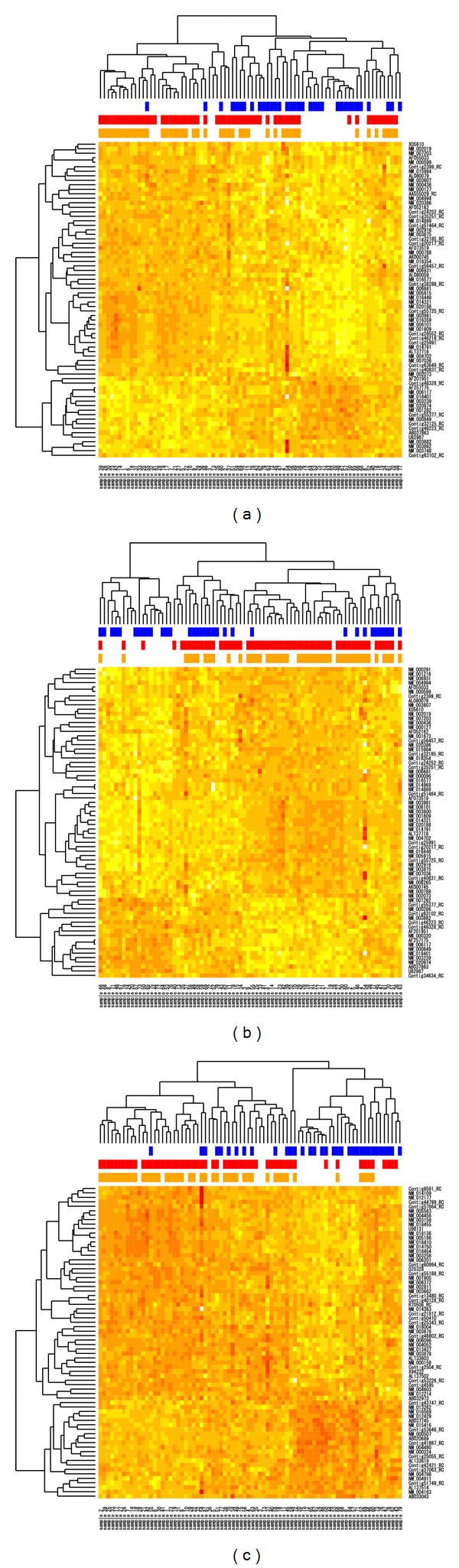
Heat maps of gene expression data with rows representing genes and columns representing patients. (a) *D*_1–70_ (MammaPrint), (b) *D*_11–80_ clearly showing some subtypes and (c) *D*_111–180_ with the highest BHI regarding metastases. The blue bars indicate patients with metastases, red bars those with ER positive and orange bars those with PR positive.

**Figure 4:**
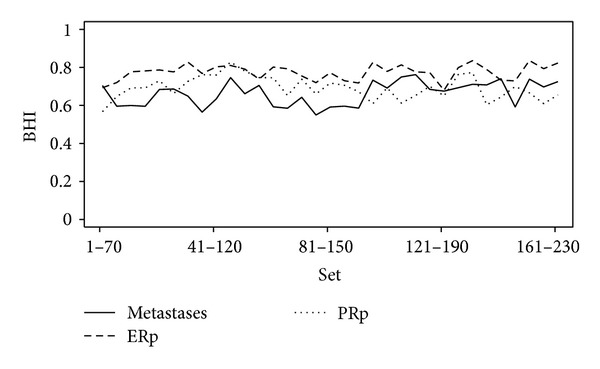
Biological homogeneity index (BHI) for metastases (solid line), ER positive (dashed line) and PR positive (dotted line).

We investigate the performance of the hierarchical clusterings based on the training data sets *D*_1–70_, *D*_11–80_ and *D*_111–180_, which are shown in Figure 3. Each row represents 70 genes and each column represents 78 patients with disease status (blue), ER status (red), and PR status (orange). The BHI for disease status, ER status, and PR status in MammaPrint (*D*_1–70_) are 0.70, 0.69, and 0.57, respectively. The gene expression in *D*_11–80_ shows different patterns from the others. Mainly, there are two clusters characterized by ER status. The left-hand side is the subgroup of patients with ER negative and poor prognosis. This would correspond to Basal-like subtype or triple negative subtype though the Her2 status is unclear. The right-hand side could be divided into three subgroups. The subgroup of patients with ER negative shows good prognosis, indicating Luminal A, and either side of it would be Luminal B or Luminal C because it shows worse prognosis than Luminal A. The data set of *D*_111–180_ has the highest BHI for disease status 0.76 and a similar gene expression pattern to that in *D*_1–70_. The other values of BHI are illustrated in Figure 4. It turned out that the data sets in *𝒟* have almost the same BHI for three statuses, suggesting there exists various gene sets with similar expression patterns.

**Figure 5:**
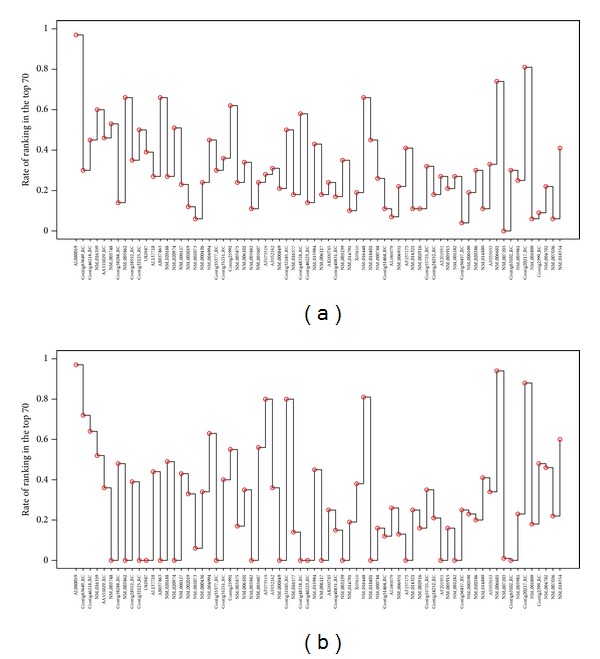
The rates of ranking in the top 70 genes by correlation coefficients (a) and by AUC (b) based on randomly sampled 50 patients. The horizontal axis denotes 70 genes used by MammaPrint.

**Figure 6:**
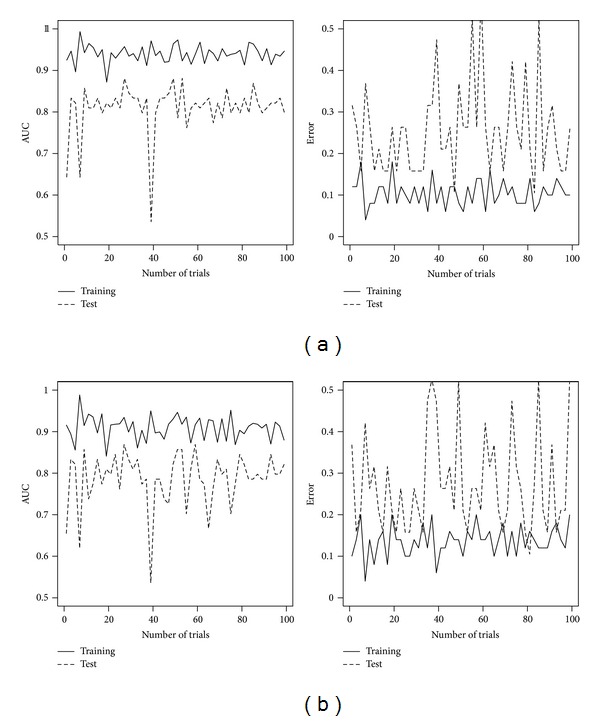
The values of AUC (left panel) and error rate (right panel) calculated by DLDA using genes ranked in top 70 by the correlation coefficients (a) and by AUC (b). These values are calculated based on randomly sampled 50 patients over 100 trials.

Next, we investigate the stability of the gene ranking in MammaPrint. Among 78 patients, we randomly choose 50 patients with 5420 gene expression patterns. Then, we take the top 70 genes ranked by the correlation coefficients of the gene expression with disease status. This procedure is repeated 100 times and checked how many times the genes in MammaPrint are ranked within the top 70. The results are shown in the upper panel of Figure 5. The lower panel shows the result based on the AUC instead of the correlation coefficient used in the ranking. We clearly see that some of the genes in MammaPrint are rarely selected in the top 70, which indicates the instability of the gene ranking. The performances of DLDA with 100 replications, which shows most stable prediction accuracy as seen in Figure 1, based on randomly selected 50 patients shown in Figure 6, where the vertical axes are AUC (left panels) and error rate (right panels) and genes are ranked by the correlation (a) and AUC (b). The performance clearly gets worse than that in Figure 1 in terms of both AUC and error rate. The heterogeneity of gene expression pattern may come from the several subtypes as suggested in the cluster analysis, and it would make it difficult to select useful genes for predictions.

**Figure 7:**
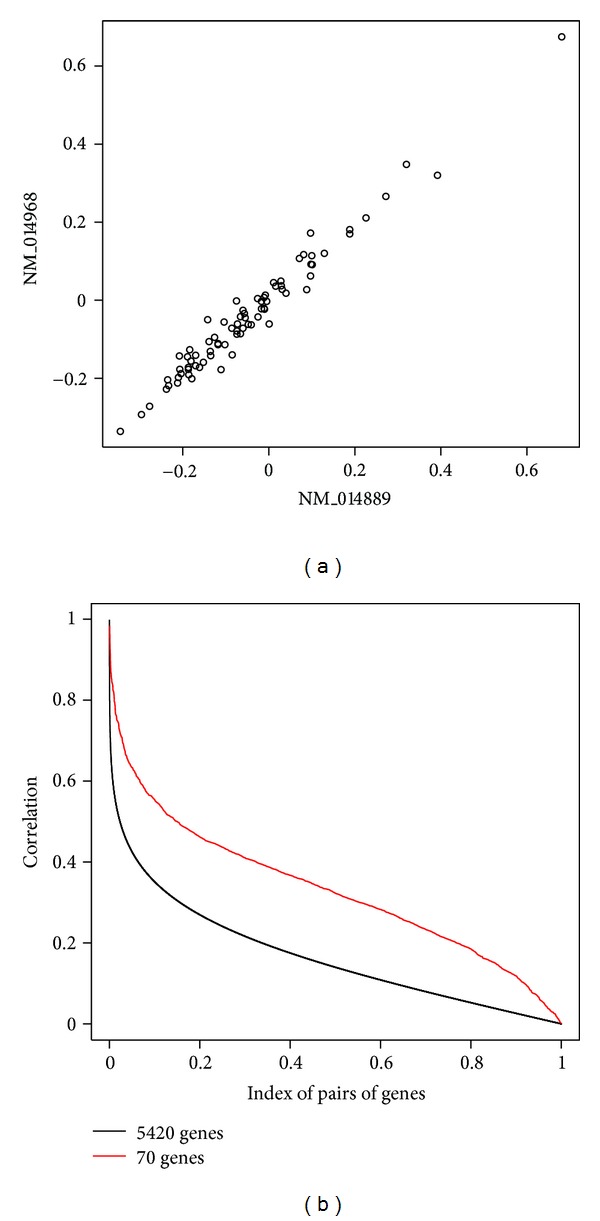
(a) Scatter plots of two pairs of genes with highest mutualcoherence (0.984) among 70 genes of MammaPrint. (b) The distribution of the correlation of total 5420 genes (black) and 70 genes of MammaPrint (red). The horizontal axis denotes the index of pairs of genes, based on which the correlations are calculated. The horizontal axis is standardized between 0 and 1 for clear view.

Finally we calculate the mutualcoherence based on genes in MammaPrint and total 5420 genes in Figure 7. The scatter plot in the upper panel (a) shows a pair of genes with the highest mutual coherence (0.984) in MammaPrint. NM_014889 and NM_014968 correspond to MP1 and KIAA1104, respectively. MP1 is a scaffold protein in multiple signaling pathways including the one in breast cancer. The latter one is pitrilysin metallopeptidase 1. The correlations defined in the right-hand side of (26) are calculated for all pairs of genes in total 5420 genes and 70 genes in MammaPrint. The distributions of them are shown in the lower panel (b) in Figure 7. The gene pairs are sorted so that the values of the correlations decrease monotonically. Note that the number of the gene pairs in each data set is different but the range of horizontal axis is restricted to 0 and 1 for clear view and for easy comparison. The difference between the black and red curves indicates that the gene set of MammaPrint has large homogeneity of gene expression patterns in comparison with that of total 5420 genes. This indicates that the ranking of genes based on two-sample statistic such as the correlation coefficients is prone to select genes such that their gene expression patterns are similar to each other. This would be one reason why we have multiple solutions after the ranking methods based on the correlation coefficients. It is also interesting to see that there are a few gene pairs with very low correlation even in gene set of MammaPrint. 

## 4. Discussion and Concluding Remarks

 In this paper we have addressed an important classification issue using microarray data. Our results present the existence of multiple suboptimal solutions for choosing classification predictors. Classification results showed that the performance of several gene sets was the same. This behavior was confirmed by the van't Veer method, DLDA, AdaBoost and AUCBoost. Amazingly, nontop ranked gene sets showed better performance than the top ranked gene sets. These results indicate that the ranking method is very unstable for feature selection in microarray data.

To examine the expression pattern of selected predictors, we performed clustering, this added support to the existence of multiple solutions. We easily recognized the good and the bad prognosis groups from the nontop ranked gene sets clustering expression pattern. Interestingly, some clustering patterns showed subtypes in the expression pattern. As described by Sørlie et al. [31] and Parker et al. [32], breast cancer could be categorized into at least five subtypes, Luminal A, Luminal B, normal breast-like, HER2, and basal-like. Selected ranked gene sets contain heterogeneous groups. Our data set does not include the necessary pathological data to categorize genes into known subtypes but considering subtypes for feature selection could be future work.

Microarray technology has become common technology and a lot of biomarker candidates are proposed. However not many researchers are aware that multiple solutions can exist in a single data set. This instability is related to the high-dimensional nature of microarray. Besides, the ranking method easily returns different results for different sets of subjects. There may be several explanations for this. The main problem is the ultra-high dimension of microarray data. This problem is known as *p* ≫ *n* problem. The ultra-high dimension gene expression data contains too many similar expression patterns causing redundancy. This redundancy is not omitted by ranking procedure. This is the critical pitfall for gene selection in microarray data.

One approach to tackle this problem about the high dimensionality of the data is to apply statistical methods in consideration with the background knowledge of medical or biological data. As seen in Figure 3 or demonstrated by Sørlie et al. [31], the subtypes of breast cancer are closely related with the resultant outcome of the patient's prognosis. This heterogeneity of data is thought as one factor that makes the ranking of the genes unstable, leading to the multiple solution for prediction rules. As seen in Figure 5 and suggested by Ein-Dor et al. [19], the gene ranking based on two-sample statistic such as correlation coefficients has large amount of variability, indicating the limitation of single gene analysis. The clustering analysis that deals with the whole information of total genes would be useful to capture the mutual relation among genes and to identify the subgroups of informative genes for the prediction problem. The combination with unsupervised learning and supervised learning is a promising way to solve the difficulty involved in the high-dimensional data.

We addressed the challenges and difficulties regarding the pattern recognition of gene expression. The main problem was caused by high-dimensional data sets. This is not only a problem of microarray data but also RNA sequencing. The RNA sequencing technologies (RNA-seq) have dramatically advanced recently and are considered an alternative to microarray for measuring gene expression as addressed in Mortazavi et al. [33], Wang et al. [34], Pepke et al. [35], and Wilhelm and Landry [36]. Witten [37] showed the clustering and classification methodology applying Poisson model for the RNA-seq data. The same difficulty occurs in the RNA-seq data. The dimension of the RNA-seq is quite high. The running cost of RNA sequencing has decreased; however, the number of features is still much larger than the number of observations. Therefore, it is not difficult to imagine that RNA-seq data would have the same difficulty as microarray. We have to be aware of it and tackle this difficulty using the insights we have learned from microarray data.

## Figures and Tables

**Table 1:** A taxonomy of feature selection techniques summarized by Saeys et al. [8]. These major feature selections are addressed. Each type has a subcategory. Advantages, disadvantages, and example methods are shown.

Model search	Advantages	Disadvantages	Examples
	*Univariate *		
Filter	Fast Scalable Independent of the classifier	Ignores feature dependencies Ignores interaction with the classifier	*χ* ^2^ Euclidean distance *t*-test Information gain
	*Multivariate *		
	Models feature dependencies Independent of the classifier Better computational complexity than wrapper methods	Slower than univariate techniques Less scalable than univariate techniques Ignores interaction with the classifier	Correlation-based feature selection Markov blanket filter Fast correlation-based feature selection

	*Deterministic *		
Wrapper	Simple Interacts with the classifier Models feature dependencies Less computationally intensive than randomized methods	Risk of overfitting More prone than randomized algorithms to getting stuck in a local optimum Classifier dependent selection	Sequential forward selection Sequential backward selection
	*Randomized *		
	Less prone to local optima Interacts with the classifier Models feature dependencies	Computationally intensive Classifier dependent selection Higher risk of overfitting than deterministic algorithms	Simulated annealing Randomized hill climbing Genetic algorithms Estimation of distribution algorithms

Embedded	Interacts with the classifier Better computational complexity than wrapper methods Models feature dependencies	Classifier dependent selection	Decision trees Weighted naive Bayes RFE-SVM
